# Computer-aided detection for prostate cancer diagnosis based on magnetic resonance imaging: Protocol for a systematic review and meta-analysis

**DOI:** 10.1097/MD.0000000000016326

**Published:** 2019-07-19

**Authors:** Fuxiang Liang, Meixuan Li, Liang Yao, Xiaoqin Wang, Jieting Liu, Huijuan Li, Liujiao Cao, Shidong Liu, Yumeng Song, Bing Song

**Affiliations:** a The First Hospital of Lanzhou University; b School of Public Health; c Evidence-Based Medicine Center, School of Basic Medical Sciences, Lanzhou University, Lanzhou; d Chinese Medicine Faculty of Hong Kong Baptist University, Hong Kong; e The First Clinical Medical College of Lanzhou University, Lanzhou; f Medical college of Soochow University, Soochow University, Suzhou; g The Second Hospital of Lanzhou University, Lanzhou, China.

**Keywords:** computer-aided detection, diagnostic accuracy, magnetic resonance imaging, meta-analysis, prostate cancer, protocol

## Abstract

**Background:**

Prostate cancer (PCa) is one of the most common primary malignancies in humans and the second leading cause of cancer-specific mortality among Western males. Computer-aided detection (CAD) systems have been developed for accurate and automated PCa detection and diagnosis, but the diagnostic accuracy of different CAD systems based on magnetic resonance imaging (MRI) for PCa remains controversial. The aim of this study is to systematically review the published evidence to investigate diagnostic accuracy of different CAD systems based on MRI for PCa.

**Methods:**

We will conduct the systematic review and meta-analysis according to the Preferred Reporting Items for a systematic review and meta-analysis of diagnostic test accuracy studies (PRISMA-DTA) guidelines. Cochrane library, PubMed, EMBASE and Chinese Biomedicine Literature Database will be systematically searched from inception for eligible articles, 2 independent reviewers will select studies on CAD-based MRI diagnosis of PCa and extract the requisite data. The quality of reporting evidence will be assessed using the quality assessment of diagnosis accuracy study (QUADAS-2) tool. Pooled sensitivity, specificity, and the area under the summary receiver operating characteristic (SROC) curves will be calculated to estimate the diagnostic accuracy of CAD system. In addition, we will conduct subgroup analyses according to the type of classifier of CAD systems used and the different prostate zoon.

**Results:**

This study will conduct a meta-analysis of current evidence to investigate the diagnostic accuracy of CAD systems based on MRI for PCa by calculating sensitivity, specificity, and SROC curves.

**Conclusion:**

The conclusion of this study will provide evidence to judge whether CAD systems based on MRI have high diagnostic accuracy for PCa.

**Ethics and dissemination:**

Ethics approval is not required for this systematic review as it will involve the collection and analysis of secondary data. The results of the review will be reported in international peer-reviewed journals.

**Prospero registration number:**

CRD42019132543.

## 1 Introduction

It is estimated that almost 1.3 million new cases of prostate cancer (PCa) and 359,000 associated deaths worldwide in 2018, accounting for 7.1% of the total new cancers diagnosed worldwide, ranking as the second most frequent cancer and the fifth leading cause of cancer death in men.^[1,2]^ PCa is the most frequently diagnosed cancer among men in over 1-half (105 of 185) of the countries of the world, notably in the Americas, Northern and Western Europe. And it is the leading cause of cancer death among men in 46 countries, particularly in Sub-Saharan Africa and the Caribbean.^[1,3]^

Therefore, reliable and early detection of PCa has become an important priority in the field of urologic oncology. For the past 25 years, Prostate-specific antigen (PSA) has always been the gold standard for the diagnosis of PCa, followed by transrectal ultrasound (TRUS)-guided biopsy, which has resulted in decreased PCa mortality by 20% to 30%,^[4]^ but with significant diagnostic errors in undersampling and understaging PCa and resulting in overtreatment related morbidity such as incontinence and impotence.^[5,6]^ Over the past decade, multi-parametric MR imaging (mp-MRI), has become the dominant non-invasive diagnostic tool for diagnosing and grading PCa.^[7]^ 3 Tesla mp-MRI enables detection of 50% of all PCa lesions and 80% of clinically significant lesions.^[8]^

However, one of the main limitations of the mp-MRI is that its interpretation requires experienced radiologists capable of analyzing data extracted from the different MR sequences, which may lead to high inter- and intra-reader variability in diagnosis.^[9]^ Therefore, automated and accurate PCa detection from mp-MRI sequences is of high demand for minimizing reading time, alleviating requirement for expertise in radiology reading, reducing risk of over-/under-treatment, and enabling large-scale PCa screening.

In the past decade, several computer-aided systems^[10–13]^ (CADs) have been developed for accurate and automated PCa detection and diagnosis. An increasing number of studies indicated that the CAD systems have the potential to support the radiologist by indicating suspicious regions and reducing oversight and perception errors.^[14]^ In addition, some CAD applications have been shown to be time efficient;^[15]^ However, the diagnostic test accuracy of different CAD systems is still controversial.

The aim of the study is to conduct a systematic review and meta-analysis to:

1) evaluate the diagnostic accuracy of CAD system based on MRI images of the prostate and provides a malignancy assessment;
2) determine which classifier of CAD system is superior for the diagnosis of PCa;
3) determine whether the performance of the CAD system depends on the specific regions of the prostate.

## 2 Methods

This research protocol has been developed according to the Preferred Reporting Items for Systematic reviews and Meta-Analyses Protocol (PRISMA-P),^[16]^ and we will conduct the systematic review and meta-analysis according to the Preferred Reporting Items for a Systematic Review and Meta-analysis of Diagnostic Test Accuracy Studies (PRISMA-DTA) guidelines.^[17]^

The protocol has been registered in PROSPERO (ID: CRD42019132543).

### 2.1 Eligibility criteria

#### 2.1.1 Types of study

We will include all studies that investigated diagnostic accuracy of CAD systems based on MRI in adult patients with suspected PCa. Included studies should have sufficient information to build a 2 × 2 contingency table (true positive [TP], false positive [FP], true negative [TN], false negative [FN]). Case–control studies will be excluded when the control group entails healthy volunteers as they are not representative of the population in which CAD will be performed.

#### 2.1.2 Participants

We will include studies that evaluate patients 18 years of age or older and with suspected PCa.

#### 2.1.3 Setting

Our study will include participants from different clinical settings, such as hospital wards, emergency departments, and intensive care units.

#### 2.1.4 Index test

We will include studies that CAD system was used to diagnose PCa, and study data was based on MRI.

#### 2.1.5 Reference standards

Biopsy should serve as the reference standard.

### 2.2 Exclusion criteria

We will exclude the studies in which the information of a 2 × 2 contingency table are lacking, and cannot be calculated from the text or appendices; and duplicated articles, review articles, editorials, case reports, summaries, animal and cell studies, meta-analysis, letters, editorials, comments, and other irrelevant article types will be also excluded.

### 2.3 Search strategy

Following databases will be systematically researched for relevant studies: Cochrane library, PubMed, EMBASE, and Chinese Biomedicine Literature Database (CBM) from their inception. There will be no restrictions placed on document language or publication status. A search strategy will be developed to define subject headings and keywords for all searches. Specific search strategies (e.g., for PubMed) are as follows: (“prostatic neoplasm∗” OR “prostate neoplasm∗” OR “prostate cancer∗” OR “prostatic cancer∗” OR “prostate tumor∗” OR “prostatic tumor∗”) AND (“artificial intelligence” OR “deep learning” OR “computer-assisted” OR “machine learning” OR “neural network∗” OR “artificial inligence” OR “AI” OR “computational intelligence” OR “machine intelligence” OR “computer reasoning” OR “automated”) AND (“diagnosis” OR “diagnos∗” OR “detection” OR “sensitivity” OR “specificity” OR “accuracy”, “positive likelihood” OR “negative likelihood” OR “ROC”).We will also contact leading authors and experts in the field of PCa for additional studies via email. The bibliographies of relevant reviews and included studies will be used to identify additional references for review. Finally, we will transfer all relevant titles and abstracts to Endnote Web for selection.

### 2.4 Study selection

**Figure 1:**
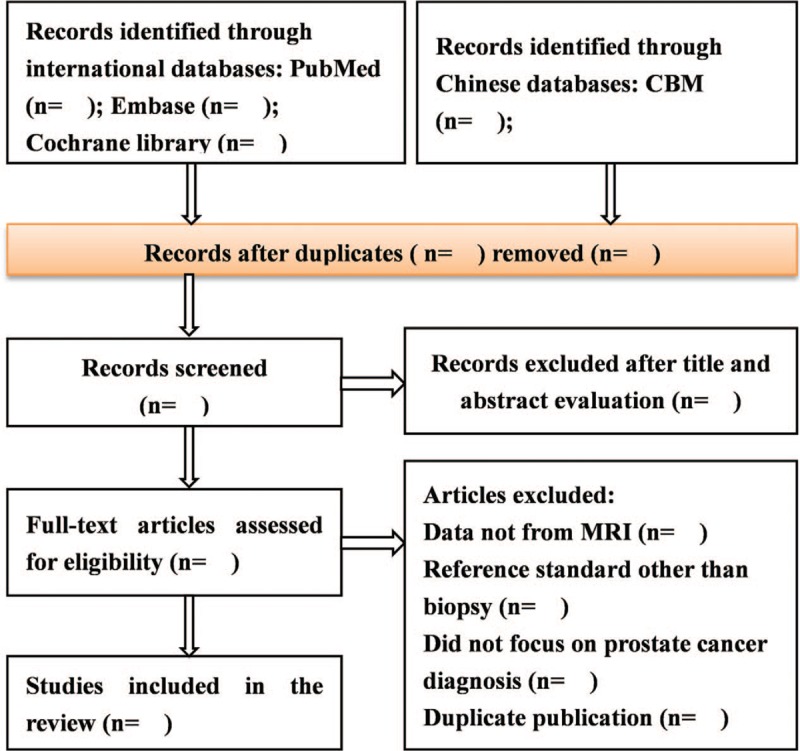
Preferred reporting items for systematic reviews and meta-analyses flow chart of study selection process.

After the removal of duplicate results, the selection of potential articles reviews will be done first by title and then by abstract by 2 independent authors (LMX and CLJ). At this stage, we will exclude studies that were not described as CAD for PCa diagnosis based on MRI. Then, the full text of each potential study will be assessed for inclusion. Disagreements will be resolved through discussion and consensus, or by consulting a third member (L-Y) of the review team. The details of study selection process will be presented in the PRISMA flow chart (shown in Fig. 1).

### 2.5 Data extraction

According to the characteristics of included studies, 2 reviewers will independently extract the following information:

Basic characteristics of included studies: first author, year of publication, country, patient numbers, patient ages, study design, PSA (ng/ml), testing set, reference standard;
The details of different CAD systems: field strength, classifier, Steps of CAD System, Imaging sequence used in system.
Diagnostic data: true positive (TP), TN, FP, FN, Accuracy, Sensitivity, and Specificity.
If there are any discrepancies, they will discuss and resolve by consensus with a third reviewer.

### 2.6 Methodological quality assessment

Two authors (LMX and LHJ) will independently evaluate the methodological quality of each eligible study using the quality assessment of diagnosis accuracy study (QUADAS-2) tool;^[18]^ discrepancies will be discussed and resolved by consensus with a third reviewer (YL). The tool is a newly revised quality assessment tool developed specifically for the systematic review of diagnostic accuracy studies, which comprises 4 domains: patient selection, index test, reference standard, flow, and timing. Each is assessed in terms of risk of bias and the first 3 in terms of concerns regarding applicability. Signaling questions are included to assist in judgments about risk of bias. And each question is answered with “yes”, “no”, “unclear”, the level of risk of bias can be judged as “low risk” “high risk” “unclear risk” homologous. Finally, Review Manager 5.3 software will be used to evaluate the risk of bias of each included study and draw the risk of bias’ figure.

### 2.7 Quality of the evidence

A Grading of Recommendations Assessment, Development and Evaluation (GRADE) approach for diagnostic tests has now been developed, which provides guidance on how to translate accuracy data into a recommendation involving patient-important outcomes.^[19]^ We will apply the GRADE approach to rate the quality of the evidence.

### 2.8 Data analysis

We will first extract the 2 × 2 contingency table (TP, FP, TN, FN). Some of the primary studies did not directly give all the data in the 2 × 2 tables, we will calculate the missing data based on the existing data in the text or appendices in each primary study using the calculator in Review Manager 5.3. Using these tables, we determined the true-positive rate (TPR; sensitivity) the true-negative rate (TNR; specificity), a descriptive forest plot and summary receiver operating characteristic (SROC) curves will be derived by Review Manager 5.3. And the stata12.0 software will be also used to develop forest plot so as to present the sensitivity and specificity and their pooled results. SROC curves are defined by sensitivity (y-axis) and specificity (x-axis), respectively, and each data point represents 1 particular study, and the area under the curve (AUC) is the final comparison indicator. The criteria for AUC classification are 0.90 to 1 (excellence), 0.80 to 0.90 (good), 0.70 to 0.80 (fair), 0.60 to 0.70 (poor), and 0.50 to 0.60 (failure).^[20]^

### 2.9 Assessment of heterogeneity

Initially, to examine heterogeneity, we will visually inspect forest plots of each study's sensitivities and specificities as well as ROC curves related to the individual study results. Statistical heterogeneity will be evaluated informally from forest plots of the study estimates and more formally using the χ2 test (*P* <.1, significant heterogeneity) and I^2^ statistic (I^2^ >50% = significant heterogeneity). In addition, different diagnostic thresholds of included studies may lead to heterogeneity; we will use the Spearman correlation coefficients to test whether there is a threshold effect. When there is a threshold effect, sensitivity and specificity will be negatively correlated, and the results will present a “shoulder-arm” point distribution on the SROC curve.

### 2.10 Subgroup analysis

We will conduct subgroup analyses according to:

a) the type of classifier of CAD systems used to determine which classifier of CAD system is superior for the diagnosis of PCa;
b) The specific regions of the prostate (peripheral zone, transitional zone, and central gland), to investigate whether the CAD diagnostic accuracy depends on the prostate zoon.

### 2.11 Assessment of publication bias

If a sufficient number of studies are identified, we will investigate publication biases by Deek's funnel plot.^[21]^ We will interpret publication bias with care because this test lacks statistical power, and adequate methods to detect publication bias in diagnostic test accuracy reviews have not been agreed on.

### 2.12 Patient and public involvement

Neither patients nor public got involved.

## 3 Discussion

Although there is some evidence on the accuracy of CAD in the diagnosis of PCa, evidence is limited and was not systematically reviewed. To the best of our knowledge, this is the first study that will systematically review CAD for PCa diagnosis based on MRI. Greater scientific rigour is necessary when establishing a diagnostic strategy that represents current evidence accurately, and we will also conduct subgroup analyses according to the type of classifier of CAD systems used and the different prostate zoon.

We will conduct a systemic review of CAD system based on MRI for the diagnosis of PCa using appropriate methodologies and quality assessment tools that may feed into an evidence-based clinical practice. This will be the first systematic review to directly compare the diagnostic accuracy of CAD system based on MRI to a reference standard of PCa.

The major limitation is that the results from this systematic review will be highly dependent on the quality of the underlying primary studies, which will be mainly retrospective studies. Another possible limitation of this study, is its susceptibility to publication and small sample biases, and may not be generalisable to other settings.

## Author contributions

**Data curation:** Meixuan Li, Huijuan Li.

**Formal analysis:** Xiaoqin Wang, Huijuan Li, Yumeng Song.

**Funding acquisition:** Jieting Liu.

**Methodology:** Fuxiang Liang, Meixuan Li, Liang Yao.

**Project administration:** Bing Song.

**Resources:** Jieting Liu.

**Software:** Meixuan Li.

**Writing – original draft:** Fuxiang Liang, Meixuan Li.

**Writing – review & editing:** Fuxiang Liang, Meixuan Li, Liang Yao, Liujiao Cao, Shidong Liu, Bing Song.
